# Independent validation of the Mosamatic deep learning automated skeletal muscle and adipose tissue segmentation tool in an external Chinese cancer patient cohort

**DOI:** 10.1093/bjrai/ubaf021

**Published:** 2026-02-24

**Authors:** Chenfei Zhou, Xuekun Zhang, David P J van Dijk, Sander S Rensen, Jun Zhang, Leroy Volmer, Leonard Wee, Steven W M Olde Damink

**Affiliations:** Department of Oncology, Ruijin Hospital, Shanghai Jiao Tong University School of Medicine, Shanghai, 200025, China; Department of Surgery, NUTRIM Institute of Nutrition and Translational Research in Metabolism, Maastricht University, Maastricht, 6200 MD, The Netherlands; Department of Radiology, Ruijin Hospital, Shanghai Jiao Tong University School of Medicine, Shanghai, 200025, China; Department of Surgery, NUTRIM Institute of Nutrition and Translational Research in Metabolism, Maastricht University, Maastricht, 6200 MD, The Netherlands; Department of Surgery, NUTRIM Institute of Nutrition and Translational Research in Metabolism, Maastricht University, Maastricht, 6200 MD, The Netherlands; Department of Oncology, Ruijin Hospital, Shanghai Jiao Tong University School of Medicine, Shanghai, 200025, China; Department of Radiation Oncology (MAASTRO), GROW Research Institute of Oncology and Reproduction, Maastricht University Medical Centre+, Maastricht, 6200 MD, The Netherlands; Department of Radiation Oncology (MAASTRO), GROW Research Institute of Oncology and Reproduction, Maastricht University Medical Centre+, Maastricht, 6200 MD, The Netherlands; Department of Surgery, NUTRIM Institute of Nutrition and Translational Research in Metabolism, Maastricht University, Maastricht, 6200 MD, The Netherlands; Department of General, Visceral and Transplantation Surgery, University Hospital Essen, Essen, 45147, Germany

**Keywords:** deep learning, automated segmentation, computed tomography, body composition

## Abstract

**Objectives:**

Deep learning neural network (DLNN)-based tools can automate body composition analysis for cancer cachexia research. We aimed to evaluate a DLNN tool trained on a European population of Chinese cancer patients.

**Methods:**

Computed tomography (CT) images at the 3rd lumbar vertebral (L3) level of Chinese gastric cancer patients were retrospectively collected. An externally validated DLNN tool (Mosamatic) was used to segment skeletal muscle, visceral adipose tissue (VAT), and subcutaneous adipose tissue (SAT). Manual segmentation was performed using SliceOmatic software (TomoVision, version 5.0). Geometric similarity between automated and manual segmentation, and the reliability was assessed.

**Results:**

The cohort comprised 203 patients with a median body mass index (BMI) of 22.2 kg/m^2^, and 604 CT images at L3 were collected. The median Dice Similarity Coefficient (IQR) of skeletal muscle, VAT and SAT were 0.973 (0.961-0.980), 0.980 (0.964-0.989), and 0.967 (0.945-0.977), respectively. The median Lin’s Concordance Correlation Coefficient for skeletal muscle area (0.983), VAT area (1.000), SAT area (0.998), skeletal muscle radiation attenuation (0.995), VAT radiation attenuation (0.994), and SAT radiation attenuation (0.997) demonstrated excellent reliability. Low BMI (<18.5 kg/m^2^) and ascites impaired the agreement between the 2 methods. The automated method showed high diagnostic concordance with manual segmentation for sarcopenia (*κ *= 0.843, *P *< .001) and myosteatosis (*κ *= 0.946, *P *< .001).

**Conclusions:**

The Mosamatic tool displays excellent generalizability to analyse body compositions in Chinese gastric cancer patients and can facilitate cachexia research.

**Advances in knowledge:**

The Mosamatic tool displayed excellent generalizability without recalibration to analyse body composition on the 3rd lumbar vertebral CT images in Chinese gastric cancer patients.

## Introduction

Body composition analysis based on computed tomography (CT) imaging is one of the fundamental methods for cancer cachexia research. Measurements obtained from the CT images at the middle level of the 3rd lumbar vertebra (L3) are validated as reliable indicators for quantitative and qualitative analysis of patients’ adipose tissue and skeletal muscle.^1^^,^^2^ Pathological alterations in body composition such as sarcopenia and myosteatosis have emerged as significant prognostic biomarkers in cancer patients, which indicates the important role of body composition analysis in cancer care management.^3^^,^^4^

Manual segmentation of skeletal muscle and adipose tissue on CT images using specialized software is one of the widely used methods for body composition analysis which demonstrates excellent inter- and intra-observer reliability. However, the labour-intensive nature and substantial time requirements limit its feasibility in routine clinical practices.^5^^,^^6^ Rapid progress in deep learning neural networks (DLNNs) enables the development of automated segmentation approaches.^7–9^ These automated tools can perform a high-throughput and reproducible analysis for large populations which facilitates the integration of body composition analysis into various clinical applications, including risk stratification, treatment decision-making, and patient follow-up.^10^^,^^11^

The accuracy and reliability of DLNN tools for radiological diagnosis are fundamentally dependent on their training datasets. Most of the existing automated tools have been developed using datasets from local regions in which the population has similar characteristics of body composition due to genetic similarity, shared lifestyle and dietary patterns.^12^^,^^13^ However, significant variations in body composition features across different ethnic groups have been observed. Specifically, the distribution and proportion of adipose tissues between Asian and Caucasian populations are distinct.^14^^,^^15^ These physiological differences may compromise the reproducibility and generalizability of a pretrained DLNN tool across diverse ethnic populations. Additionally, technical variables such as imaging configurations of CT scanner manufacturers may also influence the performance of established DLNN tools.

Therefore, assessing the reliability of a pertained DLNN-based tool in ethnically distinct populations with varying sociocultural backgrounds can help to optimize its algorithm and broaden its clinical application. Gastric cancer is one of the leading causes of cancer-related death in China, accounting for over 40% of newly diagnosed cases worldwide. Malnutrition is a common comorbidity due to tumour-related symptoms. Based on different definitions, the prevalence of sarcopenia can reach up to 60% in gastric cancer patients, and myosteatosis ranges from 24.9% to 88.9%, that are higher than other common cancers like colorectal cancer and lung cancer.^16–18^ Both sarcopenia and myosteatosis are closely associated with treatment outcomes and survival of gastric cancer patients.^19^ Given the substantial clinical need for body composition assessment, gastric cancer patients can be a suitable validation cohort.

In the present study, we aim to evaluate the reliability of an externally validated DLNN tool called Mosamatic which was developed based on Western European populations of mostly Caucasian ethnicity from the Netherlands, the United Kingdom, and Germany and shows high accuracy of body composition segmentation in patients with diverse disease conditions^20^ in an independent cohort of Chinese gastric cancer patients.

## Methods

### Patients and dataset

Clinical characteristics of 203 Chinese gastric cancer patients treated at the Department of Oncology of a Chinese hospital from 2017 to 2022 were reviewed, including age, height, weight and occurrence of ascites. Body mass index (BMI) of each patient was calculated, and the Chinese BMI criterion was used to stratify patients (underweight, BMI < 18.5 kg/m^2^; normal weight, BMI 18.5-23.9 kg/m^2^; overweight, BMI 24.0-27.9 kg/m^2^; obesity, BMI ≥ 28.0 kg/m^2^). A total of 604 abdominal CT examinations of those patients obtained at diagnosis and during treatment were reviewed, and Digital Imaging and COmmunications in Medicine (DICOM) single slices at the L3 level were collected for analysis. For contrast-enhanced CT scans, images of the portal venous phase were selected. A minority of non-contrast CT scans (2.5%, 15/604) were also included. CT examinations were performed by using equipment from 3 manufacturers including GE Medical Center, SIEMENS, and United Imaging Healthcare (UIH). The median peak voltage of the CT scan was 120 kV (80-140 kV), and the average tube current was 188 ± 96 mA.

### The DLNN tool and body composition segmentation

The pretrained DLNN tool implemented as Mosamatic was used to automatically segment skeletal muscle, visceral adipose tissue (VAT), and subcutaneous adipose tissue (SAT) on CT images (https://mosamatic.rbeesoft.nl/wordpress/, the software can be downloaded for free). No manual intervention is allowed for users. It automatically finds and annotates muscle and fat tissue in the L3 image. Muscle tissue will be assigned the label 1. Visceral fat tissue will be assigned the label 5, and subcutaneous fat tissue will get the label 7. This is according to the labelling system of the Alberta Protocol. These labels will also get their own fixed colours.

The development and validation of the DLNN tool had been described in a previous report.^20^ In brief, the DLNN consisted of 2 independently trained DLNNs, both derived from a canonical 2D U-net deep learning network. The first DLNN was trained to extract the abdomen only, thereby excluding hands, arms, CT bed and external devices that may be present in the image. The second DLNN was trained to perform the actual segmentation of muscle, VAT and SAT only within the abdominal contour extracted in the first DLNN. The DLNN produced highly concordant results in an independent external validation cohort of Scottish patients.

### Reference segmentation

A trained analyst manually segmented the CT images by using SliceOmatic software (TomoVision, version 5.0) following the Alberta Protocol as the standard reference. Predefined Hounsfield unit (HU) ranges of skeletal muscle (−29 to 150 HU), VAT (−150 to −50 HU), SAT (−190 to −30 HU), and intramuscular adipose tissue (IMAT, −190 to −30 HU) were used.^21^ Due to the heterogenous distribution of IMAT, it can hardly be assessed accurately based on a single CT scan, and its clinical significance is still under investigation. Mosamatic tool is not designed to analyse IMAT during its development and training. Therefore, IMAT was excluded from analysis. Segmented images were independently checked by an experienced author. Areas of skeletal muscle, VAT and SAT were recorded from the human-expert segmentations, as well as from the output of the Mosamatic tool. The skeletal muscle index (SMI) was calculated using the skeletal muscle area divided by the height of the patients squared. Mean radiation attenuation values of skeletal muscle (SMRA), visceral adipose tissue (VATRA), and subcutaneous adipose tissue (SATRA) were calculated. Diagnosis of sarcopenia (male: BMI < 25.0 kg/m^2^ and SMI < 43cm^2^/m^2^ or BMI ≥ 25.0 kg/m^2^ and SMI < 53cm^2^/m^2^; female: SMI < 41cm^2^/m^2^) and myosteatosis (BMI < 25.0 kg/m^2^ and SMRA < 41HU or BMI ≥ 25.0 kg/m^2^ and SMRA < 33HU) were performed following the previously reported cut-offs.^22^

### Statistical analysis

The geometric similarity between the automated tool and manual segmentation was analysed using a Dice Similarity Coefficient (DSC) score, and 3 indices including skeletal muscle, VAT and SAT were compared. Perfect agreement implied a DSC score of 1, whereas no overlap at all between the reference and automated segmentation implied a DSC score of 0. Agreement of body composition indices was quantified using Lin’s concordance correlation coefficient (CCC), intraclass correlation coefficient (ICC), and limits of agreement interval (LOA) of Bland-Altman plot. Bland-Altman and Lin’s CCC analysis was performed taking into account replicated measures per subject, as described by Parker et al,^23^ which uses the R package “nlme.”^24^ Perfect agreement implied a CCC of 1. LOA intervals of agreement were close to 0 which implied perfect agreement. Proportional bias of LOA was analysed using a 1-sample *t* test. Difference of DSC score of skeletal muscle, VAT and SAT among clinical factors (gender, BMI group, and ascites) was analysed using independent-samples *t* test and 1-way ANOVA test (Least Significant Difference method). Concordance of sarcopenia and myosteatosis diagnosis between the automated tool and manual segmentation was analysed using Cohen’s *κ* coefficient. *P* values less than .05 were considered statistically significant. Data analyses were performed by SPSS 22.0 software (SPSS Inc., Chicago, IL, United States).

## Results

### Demographic information and body composition indices of patients

The CT examinations from 203 gastric cancer patients (132 male and 71 female) with a median age of 62 years were reviewed. The median body mass index of the cohort was 22.2 kg/m^2^, and 8.9% of patients were classified as underweight. Ascites occurred at diagnosis or during treatment in 15 patients (7.4%). Detailed demographic information of patients at diagnosis was listed in Table 1. From these patients, 604 CT images were collected, and the median number of CT images per patient was 3. Quantitative measurements of body composition indices including skeletal muscle area, VAT area, SAT area, and their respective radiation attenuation values (SMRA, VATRA, and SATRA) obtained through both manual and automated segmentation were listed in Table S1.

**Table 1.** Clinical characteristics of subjects.

Clinical characteristics	Total (*n* = 203)	Male (*n* = 132)	Female (*n* = 71)
Age (years) (range)	62.0 (25.0-81.0)	64.0 (26.0-78.0)	59.0 (25.0-81.0)
Height (cm) (range)	168.0 (145.0-185.0)	170.0 (155.0-185.0)	160.0 (145.0-173.0)
Weight (kg) (range)	63.0 (42.1-99.0)	65.0 (45.0-99.0)	55.0 (42.1-79.0)
BMI (kg/m^2^) (range)	22.2(13.4-34.3)	22.6 (13.4-34.3)	21.3 (17.5-32.0)
BMI group, *n* (%)			
Underweight (<18.5)	18 (8.9)	11 (8.3)	7 (9.9)
Normal (18.5-23.9)	118 (58.1)	74 (56.1)	44 (62.0)
Overweight (24.0-27.9)	55 (27.1)	38 (28.8)	17 (23.9)
Obesity (≥28.0)	12 (5.9)	9 (6.8)	3 (4.2)
Ascites, *n* (%)	15 (7.4)	7 (5.3)	8 (11.3)

Abbreviations: BMI = body mass index.

### Agreement between automated and manual segmentation

**Figure 1.**
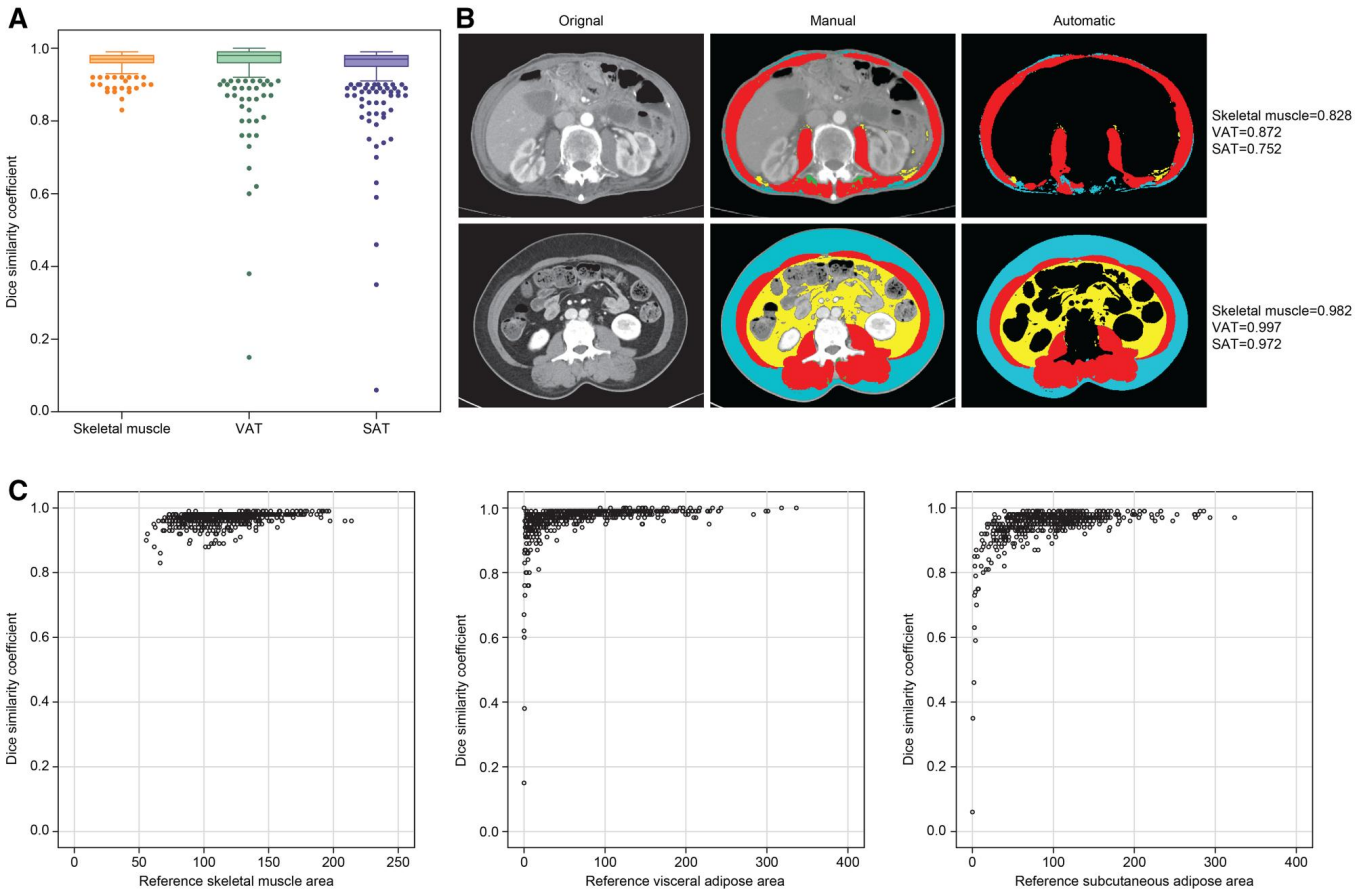
The geometric similarity between manual and automated segmentation. (A) DSC scores of skeletal muscle, VAT, and SAT of CT images. (B) Representative images of original, manual segmentation, and the automated segmentation with low and high DSC scores. (C) Distribution of DSC scores of skeletal muscle, VAT, and SAT corresponding to the area. Abbreviations: DSC = Dice Similarity Coefficient; SAT =subcutaneous adipose tissue; VAT =visceral adipose tissue.

For all 604 CT images, the median DSC scores (IQR) of skeletal muscle, VAT, and SAT were 0.973 (0.961-0.980), 0.980 (0.964-0.989), and 0.967 (0.945-0.977), respectively (Figure 1A). Representative images with high and low DSC scores are illustrated in Figure 1B. The distribution of DSC score for skeletal muscle area was better than the other 2 indices with a minimum value of 0.830. Samples with low area of skeletal muscle, SAT, and VAT tended to have a lower DSC score (Figure 1C). The extreme outliers of the DSC score for VAT and SAT were also observed and were illustrated in Figure S1.

**Figure 2.**
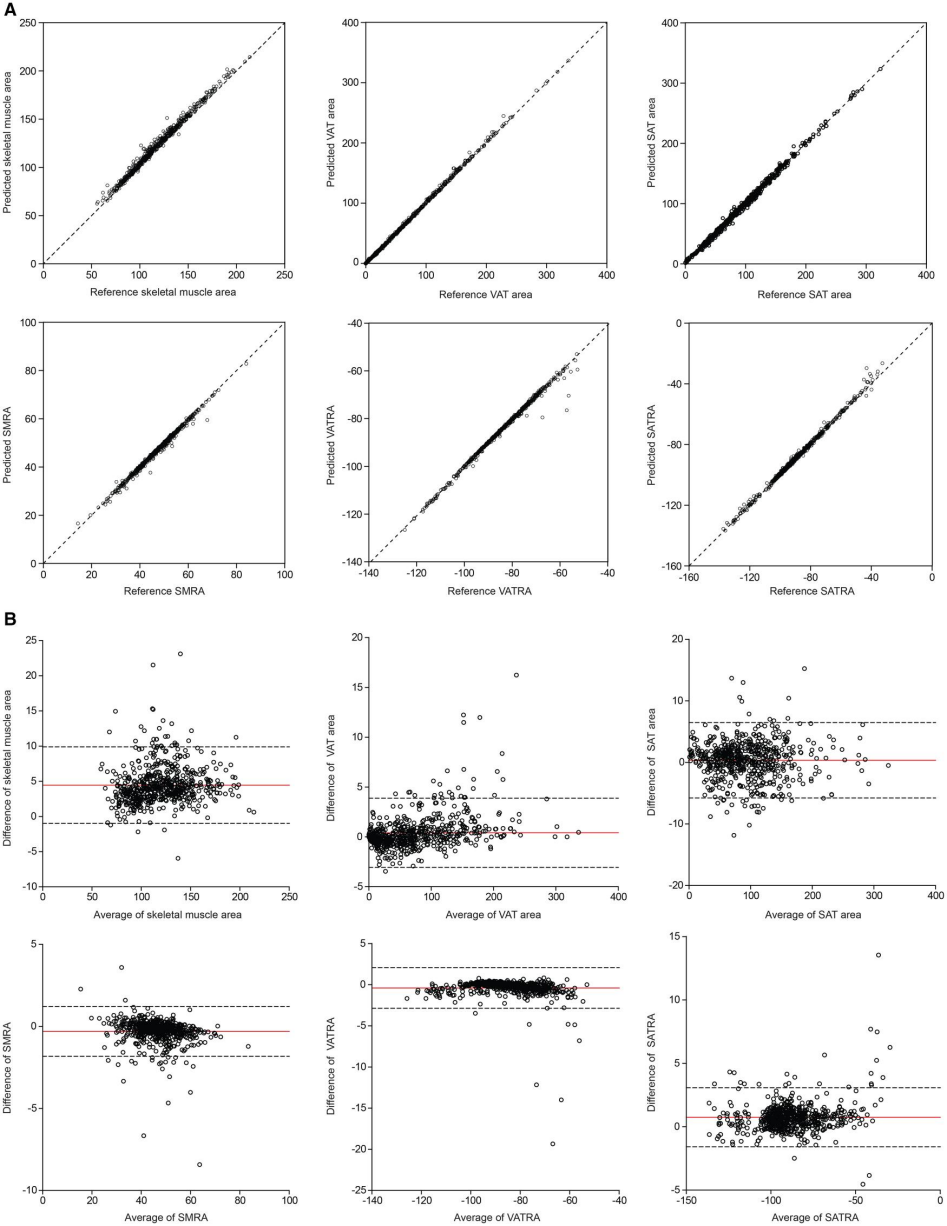
The agreement of body composition indices between manual and automated segmentation. (A) Correlation analysis of body composition indices of 2 approaches. (B) The Bland-Altman plots of the automated segmentation compared with manual segmentation.

Lin’s CCC and ICC of skeletal muscle, VAT, and SAT were all above 0.98, implying very high agreement. The detailed values are listed in Table 2, and correlation plots of these indices are illustrated in Figure 2A. The mean systematic errors (Bland-Altman as aforementioned) between segmented and expert-drawn cross-sectional areas were small and statistically non-significantly divergent from zero: 4.4 cm^2^ (95% CI, −1.0 to 9.9), 0.38 cm^2^ (95% CI, −5.8 to 6.5), and 0.42 cm^2^ (95% CI, −3.1 to 3.9) for skeletal muscle, subcutaneous adipose and visceral adipose, respectively. Similarly, mean systematic errors in radiation attenuation values were -0.3 HU (95% CI, −1.8 to 1.2), 0.8 HU (95% CI, −1.6 to 3.1), and −0.4 HU (95% CI, −2.9 to 2.1) for skeletal muscle, subcutaneous adipose, and visceral adipose, respectively. For the LOA interval analysis of Bland-Altman plot, no proportional bias was observed in the SAT area. The differences between the automated and manual segmentation results of most CT images were assessed by LOA intervals (Figure 2B). The median percentages of difference for the body composition indices were 3.46% (skeletal muscle area), 0.07% (VAT area), 0.73% (SAT area), −0.39% (SMRA), 0.19% (VATRA), and −0.74% (SATRA).

**Table 2.** Correlation and agreement statistics of subjects between automated and manual segmentation.

Parameters	Lin’s CCC	ICC (95%CI)	Bland-Altman plot		
			LOA, mean (95% CI)	*P* value	Performance
Skeletal muscle area (cm^2^)	0.983 (0.981-0.985)	0.983 (0.541-0.995)	4.4 (−1.0 to 9.9)	<.001	Proportional bias
VAT area (cm^2^)	1.000 (0.999-1.000)	1.000 (0.999-1.000)	0.38 (−5.8 to 6.5)	<.001	Proportional bias
SAT area (cm^2^)	0.998 (0.998-0.998)	0.998 (0.998-0.998)	0.42 (−3.1 to 3.9)	.174	No proportional bias
SMRA (HU)	0.995 (0.995-0.996)	0.995 (0.993-0.997)	−0.3 (−1.8 to 1.2)	.001	Proportional bias
VATRA (HU)	0.994 (0.993-0.995)	0.994 (0.992-0.996)	−0.8 (−1.6 to 3.1)	<.001	Proportional bias
SATRA (HU)	0.997 (0.997-0.998)	0.997 (0.992-0.999)	−0.4 (−2.9 to 2.1)	<.001	Proportional bias

Abbreviations: ICC = Intraclass correlation coefficient; Lin’s CCC = Lin’s concordance correlation coefficient; LOA = limits of agreement interval; SCC = Spearman correlation coefficient.

### Clinical and image factors affecting geometric agreement

**Figure 3.**
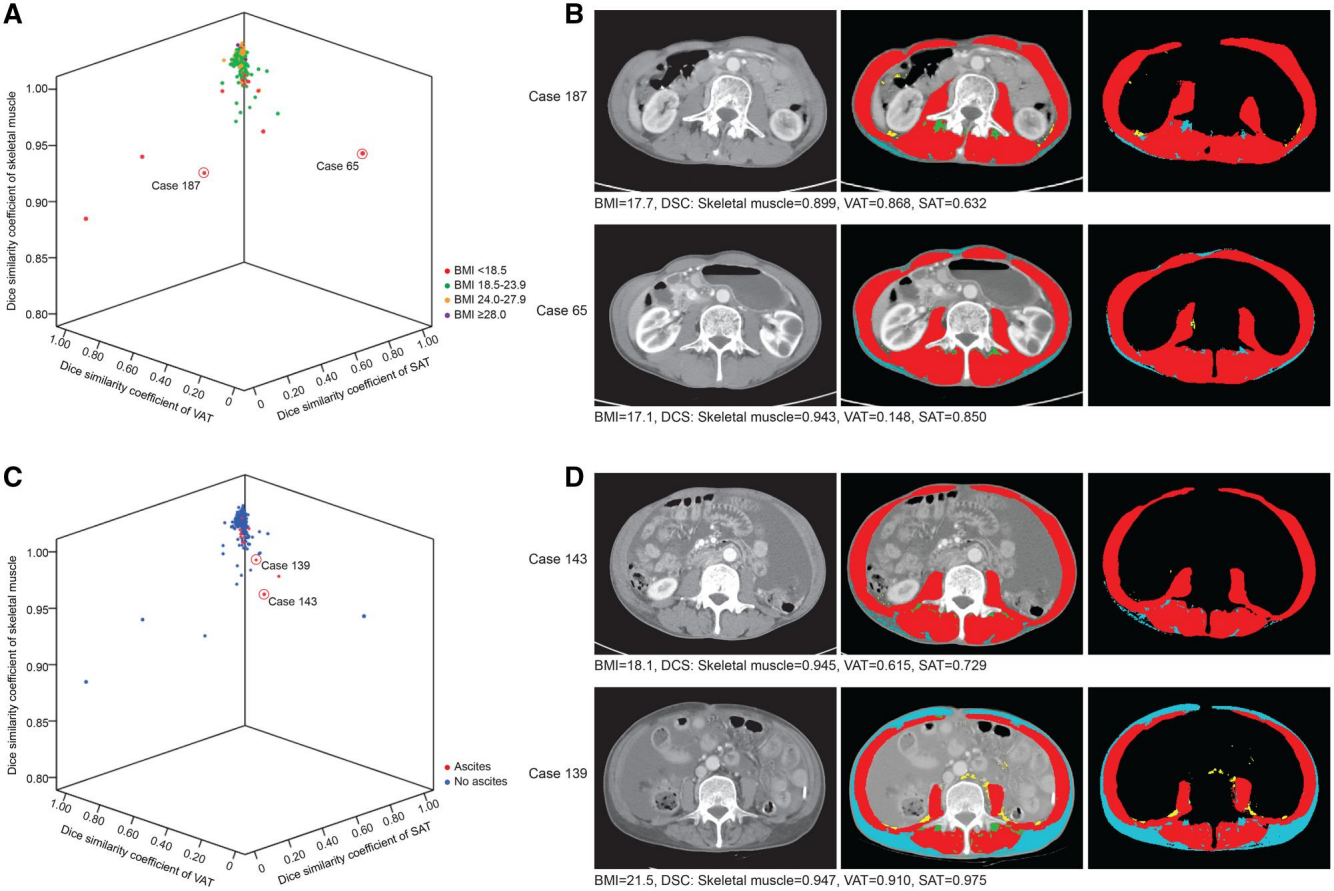
Distribution of DSC scores of body composition indices. (A) Distribution of DSC scores of body composition indices in patients with different BMI groups. (B) Representative images of subjects with low BMI. (C) Distribution of DSC scores of body composition indices in patients with or without ascites. (D) Representative images of subjects with ascites. Abbreviations: BMI =body mass index; DSC =Dice Similarity Coefficient.

The association between clinical factors and DSC scores of body composition was analysed based on CT images at diagnosis of patients (Table 3). The DSC score of SAT in female patients was higher than that in male patients (*P *= .002). The DSC scores of skeletal muscle, VAT, and SAT in patients with low BMI (<18.5 kg/m^2^) were significantly lower than those of patients in other BMI groups (Figure 3A). The DSC score of skeletal muscle in patients with ascites was lower than that of patients without ascites (Figure 3C).

**Table 3.** DSC scores of body composition indices affected by clinical factors.

Clinical factors	Skeletal muscle (IQR)	VAT (IQR)	SAT (IQR)
All (*n* = 203)	0.978 (0.971-0.982)	0.986 (0.970-0.991)	0.970 (0.952-0.979)
Male (*n* = 132)	0.978 (0.973-0.983)	0.987 (0.970-0.992)	0.965 (0.943-0.978)
Female (*n* = 71)	0.975 (0.969-0.980)	0.982 (0.970-0.990)	0.975 (0.964-0.982)^a^
BMI group			
Underweight (*n* = 18)	0.961 (0.944-0.968)^b^	0.951 (0.901-0.980)^b^	0.938 (0.780-0.972)^b^
Normal (*n* = 118)	0.971 (0.970-0.981)^c^	0.982 (0.970-0.989)	0.968 (0.954-0.978)
Overweight (*n* = 55)	0.981 (0.976-0.984)	0.990 (0.985-0.993)	0.975 (0.960-0.982)
Obesity (*n* = 12)	0.983 (0.979-0.987)	0.991 (0.988-0.995)	0.973 (0.971-0.983)
Ascites			
No (*n* = 188)	0.978 (0.973-0.982)^a^	0.987 (0.971-0.991)	0.971 (0.952-0.979)
Yes (*n* = 15)	0.968 (0.961-0.978)	0.970 (0.955-0.988)	0.964 (0.948-0.982)

a The *P* values of DSC scores of body composition indices were <.05 compared with the other groups.

b The *P* values of DSC scores of body composition indices in underweight group were <.05 compared with all other 3 BMI groups.

c The *P* value of DSC score of skeletal muscle in normal weight group was <.05 compared with all other 3 BMI groups.

Images with outliers of the DSC score of body composition were further reviewed. Among 66 images with low DSC score (<0.9) in either skeletal muscle, SAT, or VAT, the main reasons for outliers of DSC score were low SAT area (59.1%), low VAT area (31.8%), misjudgement of SAT and IMAT (19.7%), abnormal density in SAT (10.6%), ascites (6.1%), misjudgement of skeletal muscle and adjacent organs (4.5%), and image noise (4.5%) (Table S3).

### Concordance of sarcopenia and myosteatosis diagnosis

The values of SMI and SMRA at diagnosis were used to identify sarcopenia and myosteatosis in patients. High concordance between the results of manual and automated segmentation was observed (Table S2). The diagnosis of myosteatosis by 2 approaches was almost the same (*κ *= 0.946, *P *< .001; sensitivity 0.97, specificity 0.99), as well as for sarcopenia diagnosis (*κ *= 0.843, *P *< .001; sensitivity 0.86, specificity 1.00).

## Discussion

In this study, the DLNN automatic segmentation tool Mosamatic showed excellent geometric agreement with manual segmentations of CT images in an independent cohort of Chinese gastric cancer patients. Low BMI and ascites are the clinical factors which impaired geometric similarity. The high concordance for the diagnosis of sarcopenia and myosteatosis using results of automated and manual segmentation can be observed.

The automated tool applied in our study was trained based on 3413 abdominal cancer surgery subjects collected from 31 medical centres located in the United Kingdom, the Netherlands, and Germany, and was externally tested in an independent UK cohort.^20^^,^^25^ Its high accuracy was further independently validated in a large polytrauma registry dataset of Western patients and showed the median DSC scores of skeletal muscles, VAT, and SAT were 0.97, 0.97, and 0.96, respectively.^26^ Several automated segmentation approaches based on neural network methods have also been developed by other groups. The DSC scores of these approaches are comparable to our tool with a range from 0.930 to 0.983 for skeletal muscle, 0.915 to 0.979 for VAT, and 0.980 to 0.986 for SAT.^7^^,^^9^^,^^27^^,^^28^

External validation of a trained automated tool in an ethnic population with a different sociocultural background is barely reported. Although multiple large external validation cohorts with various diseases including polytrauma, colorectal, ovarian, and pancreatic cancer were used during the training and development of our automated tool, these datasets predominantly consisted of Western populations. In this study, the results demonstrate that Mosamatic tool performs excellently in Chinese gastric cancer patients. The mean DSC scores for skeletal muscle, VAT, and SAT all exceed 0.95, which are comparable to the results of previous validation cohorts. Notably, the subjects evaluated in the present study have lower median weight, height, and BMI compared with the European subjects enrolled in the training stage of our automated tool, while scanner manufacturers are similar.^25^

The excellent agreement of both area and radiation attenuation of body composition indices results in a high concordance of sarcopenia and myosteatosis diagnosis. The myosteatosis rate of gastric cancer patients diagnosed using SMRA values detected by automated and manual segmentation is nearly identical, with a sensitivity of 0.97 and specificity of 0.99. Body composition indices, particularly the index and radiation attenuation of skeletal muscle, have been identified as prognostic biomarkers of cancer patients treated with diverse therapeutics such as surgery, chemotherapy, and immunotherapy.^18^^,^^29^^,^^30^ The results of the present study suggest the feasibility of using the Mosamatic automated tool in clinical practices of both Western and Asian international cohorts.

The median percentage of difference for skeletal muscle area between manual and automated segmentation was relatively higher than other indices. The difference exhibited high consistency across images which suggested a system offset. The judgement of the analyst during manual segmentation serving as a reference could be one of the reasons. Manual segmentation tends to leave a margin for adjacent tissues and avoid to tag tissues with similar HU with skeletal muscle, such as skin, muscle fascia, surgical scar, and hypoproteinaemia-related subcutaneous oedema. In our study, abnormal density observed within SAT and misjudgement of skeletal muscle with adjacent organs are the major reasons for segmentation failure for skeletal muscle, which could contribute to misclassification by the DLNN.^31^ Anatomic variations of skeletal muscles of the abdominal wall at the L3 level, shown on a CT scan, can also influence the accuracy of automated segmentation.^32^^,^^33^

Currently, some novel algorithms have been developed to improve the accuracy of skeletal muscle segmentation. A 3D site-specific segmentation of skeletal muscles by learning 2 muscle regions using a 2D U-Net can achieve a higher accuracy of skeletal muscle segmentation within the L3 slice.^34^ A joint segmentation using multiclass learning with a 2D U-Net architecture which incorporates knowledge from the surrounding muscle context enhances the performance of site-specific segmentation.^35^ Kamiya et al developed an automated segmentation method for surface muscles using a 3D U-Net based on selective voxel patches for whole-body CT images which can segment surface muscles from limited annotation data.^36^ The combination of site-specific and whole-body skeletal muscle recognition can be an effective strategy to improve the performance of our tool.

Although the difference in skeletal muscle segmentation between the manual and automatic methods led to a potential underestimation of sarcopenia diagnosis, the mean difference of skeletal muscle area of those unmatched patients was only 1.95 cm^2^. Such marginal deviation could also be observed among experienced analysts performing manual segmentation.^26^ For the diagnosis of sarcopenia and myosteatosis, current criteria based on a single parameter can result in controversy for borderline cases. Therefore, a novel criterion using multiple parameters combination and dynamic detection might be developed to provide accurate diagnosis.

In this study, low BMI (<18.5 kg/m^2^) and the presence of ascites were associated with lower DSC scores. The DSC metric is known to be overly sensitive to small volume of regions. Therefore, patients with extremely low volume of adipose tissue show outliers in DSC scores for VAT and SAT, which are the main reasons for segmentation failures. However, it is important to note that DSC score is not the only criterion for judging the quality of the DLNN segmentation. It is the non-linearity in clinical significance which suggests a higher DSC score does not necessarily imply a different clinical significance compared to a lower one. Nevertheless, low BMI and severe adipose tissue wasting are common phenomena in Chinese patients with upper gastrointestinal cancer who had high prevalence of cancer cachexia. For the DLNN tool, it learns the most from the middle of the range and tends to work better closer to the median samples rather than the extremities. Continuous training of the DLNN tools by using massively representative and massively diverse training datasets covering all kinds of body phenotypes might help to improve the performance of our automated tool in the future. On the other hand, whole-body CT image analysis that can provide a more accurate and comprehensive assessment can be a solution for patients with these specific clinical conditions, and will be more widely used after balancing the accuracy and feasibility.^9^

The intermuscular adipose tissue (IMAT) is not assessed by our automated tool. Currently, few DLNN tools have been developed for IMAT segmentation, and show suboptimal geometric similarity, with DSC scores ranging from 0.89 to 0.92, which is substantially lower than those achieved for other body composition indices.^8^^,^^28^ Although the clinical relevance of IMAT in cancer patients has been recognized, its evaluation remains challenging.^37^^,^^38^ With the high heterogenous distribution and low content of IMAT, quantitative analysis for IMAT of the whole body is hardly performed on one slice of CT scan.^39^ Therefore, we excluded IMAT segmentation from our DLNN training protocol. Future studies should investigate whole-muscle compartments analysis as a potentially more robust assessment approach for IMAT.

There are several limitations of the present study. Only the baseline demographic information of patients was recorded which limited the sample size when evaluating the impact of clinical factors on geometric similarity. The poor quality of CT images with low contrast which could hardly be manually segmented was one of the causes of the extreme outliers of DSC scores for VAT and SAT. Despite the low amount, CT images with poor quality can be observed during clinical practices. Therefore, we kept these results in the present study which could reflect the real-world data. By continuous training with different CT images, the DLNN could be further improved in the future.

## Conclusion

The Mosamatic tool developed and trained based on the Western population displayed excellent generalizability to analyse body composition on L3 CT images in Chinese gastric cancer patients.

## Supplementary Material

ubaf021_Supplementary_Data

## Data Availability

The data supporting the conclusions of this study are included in the supplementary material files and available from the corresponding author on request.
