# Supplementary material for: Independent validation of the Mosamatic deep learning automated skeletal muscle and adipose tissue segmentation tool in an external Chinese cancer patient cohort
Source: BJR Artif Intell. 2026 Feb 24;3(1):ubaf021. doi: 10.1093/bjrai/ubaf021 (PMC13045518; doi:10.1093/bjrai/ubaf021)
Supplement: ubaf021_Supplementary_Data [file ubaf021_supplementary_data.zip › Supplementary files 20251031.docx]

**Title:** Independent validation of the Mosamatic deep learning automated skeletal muscle and adipose tissue segmentation tool in an external Chinese cancer patient cohort

Supplementary information:

**Table S1. Body composition indices detected by the DLNN and manual segmentation**

| Indices | Automated segmentation median (IQR) | Manual segmentation median (IQR) |
| --- | --- | --- |
| Skeletal muscle area (cm^2^) | 120.18 (99.92, 139.72) | 114.68 (96.48, 134.85) |
| VAT area (cm^2^) | 58.92 (21.21, 112.45) | 58.41 (21.34, 111.77) |
| SAT area (cm^2^) | 87.78 (57.55, 124.13) | 87.60 (56.78, 123.29) |
| SMRA (HU) | 46.30 (40.48, 51.63) | 46.50 (40.77, 52.06) |
| VATRA (HU) | -84.90 (-92.63, -76.54) | -84.84 (-92.77, -75.96) |
| SATRA (HU) | -89.63 (-97.77, -79.09) | -90.52 (-98.67, -79.55) |

**Table S2. Concordance test of sarcopenia and myosteatosis identified by automated and manual segmentation**

|  |  | Automated | | *κ* value | *P* value |
| --- | --- | --- | --- | --- | --- |
|  | Manual | Yes | No |  |  |
| Sarcopenia | Yes | 97 (85.8) | 16 (14.2) | 0.843 | <0.001 |
|  | No | 0 (0) | 90 (100) |  |  |
| Myosteatosis | Yes | 32 (97.0) | 1 (3.0) | 0.946 | <0.001 |
|  | No | 2 (1.2) | 168 (98.8) |  |  |

**Table S3 Qualitative Analysis of Segmentation Failures (n=66)**

| Reasons | Images (n) | Percentage |
| --- | --- | --- |
| Low subcutaneous adipose tissue area | 39 | 59.1% |
| Low visceral adipose tissue area | 21 | 31.8% |
| Misjudgment of subcutaneous adipose tissue and intramuscular adipose tissue | 13 | 19.7% |
| Abnormal density in subcutaneous adipose tissue | 7 | 10.6% |
| Ascites | 4 | 6.1% |
| Misjudgment of skeletal muscle and adjacent organs | 3 | 4.5% |
| Image noise | 3 | 4.5% |

**Figure S1**

A: Distribution of DSC scores of body composition indices

B: CT images with lowest DSC scores of skeletal muscle, VAT, and SAT, respectively.
