# Supplementary material for: Independent validation of the Mosamatic deep learning automated skeletal muscle and adipose tissue segmentation tool in an external Chinese cancer patient cohort
Source: BJR Artif Intell. 2026 Feb 24;3(1):ubaf021. doi: 10.1093/bjrai/ubaf021 (PMC13045518; doi:10.1093/bjrai/ubaf021)
Supplement: ubaf021_Supplementary_Data [file ubaf021_supplementary_data.zip › Figure S1.pdf]

A

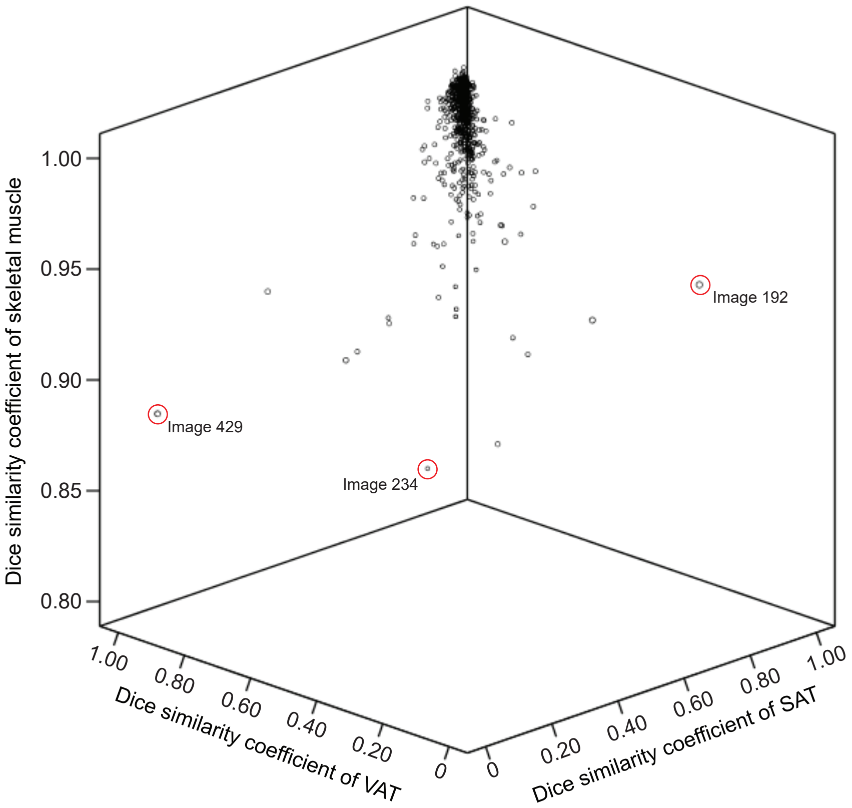

B

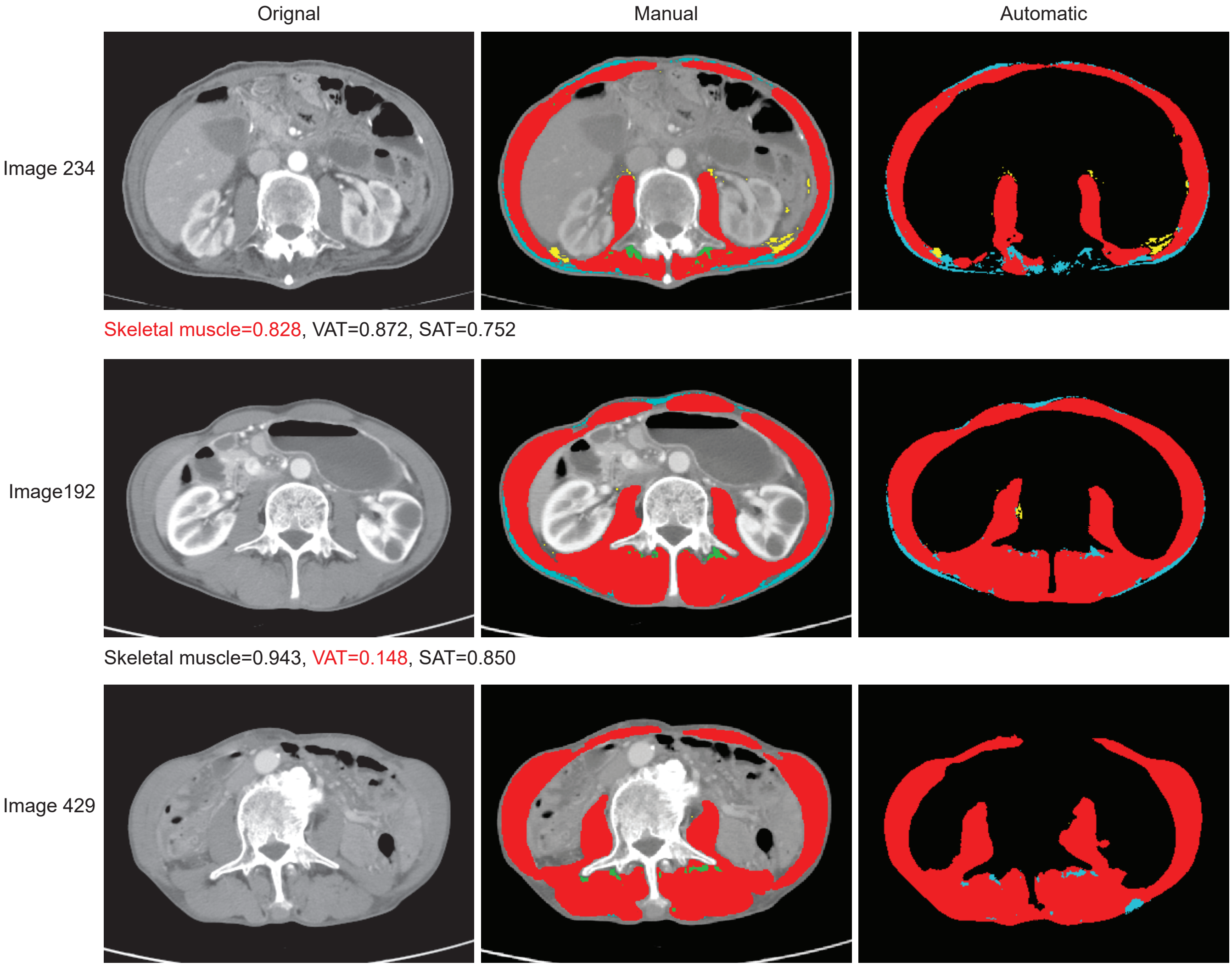

Skeletal muscle=0.828, VAT=0.872, SAT=0.752

Skeletal muscle=0.943, VAT=0.148, SAT=0.850

Skeletal muscle=0.871, VAT=1.000, SAT=0.063
